# The metabolic consequences of HIV/TB co-infection

**DOI:** 10.1186/s12879-023-08505-4

**Published:** 2023-08-18

**Authors:** Chandré Herbert, Laneke Luies, Du Toit Loots, Aurelia A. Williams

**Affiliations:** Human Metabolomics, North-West University, Potchefstroom, South Africa

**Keywords:** HIV/AIDS, Tuberculosis, HIV/TB co-infection, Metabolomics, Metabolism, GCxGC-TOFMS, Gut microbiome

## Abstract

**Background:**

The synergy between the human immunodeficiency virus (HIV) and *Mycobacterium tuberculosis* during co-infection of a host is well known. While this synergy is known to be driven by immunological deterioration, the metabolic mechanisms that contribute to the associated disease burden experienced during HIV/tuberculosis (TB) co-infection remain poorly understood. Furthermore, while anti-HIV treatments suppress viral replication, these therapeutics give rise to host metabolic disruption and adaptations beyond that induced by only infection or disease.

**Methods:**

In this study, the serum metabolic profiles of healthy controls, untreated HIV-negative TB-positive patients, untreated HIV/TB co-infected patients, and HIV/TB co-infected patients on antiretroviral therapy (ART), were measured using two-dimensional gas chromatography time-of-flight mass spectrometry. Since no global metabolic profile for HIV/TB co-infection and the effect of ART has been published to date, this pilot study aimed to elucidate the general areas of metabolism affected during such conditions.

**Results:**

HIV/TB co-infection induced significant changes to the host’s lipid and protein metabolism, with additional microbial product translocation from the gut to the blood. The results suggest that HIV augments TB synergistically, at least in part, contributing to increased inflammation, oxidative stress, ART-induced mitochondrial damage, and its detrimental effects on gut health, which in turn, affects energy availability. ART reverses these trends to some extent in HIV/TB co-infected patients but not to that of healthy controls.

**Conclusion:**

This study generated several new hypotheses that could direct future metabolic studies, which could be combined with other research techniques or methodologies to further elucidate the underlying mechanisms of these changes.

**Supplementary Information:**

The online version contains supplementary material available at 10.1186/s12879-023-08505-4.

## Background

People living with the human immunodeficiency virus/acquired immunodeficiency syndrome (PLWHA) have a 26–31 times greater risk of developing active tuberculosis (TB) compared to human immunodeficiency virus (HIV)-negative individuals [1] due to the immunosuppressive effects of HIV on the host. TB is the most common secondary disease among PLWHA, driving the global burden of HIV/TB co-infection, which has been exacerbated by diminished access to health care facilities during the coronavirus disease 2019 (COVID-19) pandemic. This resulted in poor diagnosis, reporting, and treatment initiation for HIV and TB cases, increasing TB-related deaths during this period [2–4]. Approximately 12% of the global estimated 1.6 million TB-related deaths in 2021 were among PLWHA [5].

HIV (all references to “HIV” imply HIV-1) and *Mycobacterium tuberculosis* (*Mtb*) act synergistically during co-infection of a host, amplifying the disease burden. This synergy is centered on immunological deterioration [6, 7], which is the result of an increased level of immune activation [8] and oxidative stress (OS) [9]. The immune and metabolic systems are intricately connected, and changes in one are reflected in the other. While the host undergoes metabolic changes as part of immune processes, the pathogen modulates and/or reprograms host metabolism to suit its own needs, for example, reproduction and immune evasion or persistence [10]. This leads to metabolic dysfunction in the host, a condition that becomes particularly prominent if the immune response fails to eliminate the pathogen and the infection becomes chronic. This is the result of a continuously active immune response, forcing the host to adapt to long periods of higher energy and biosynthetic demand [11].

Metabolic alterations during co-infection are not well characterized. However, conventional techniques relying on low-throughput and laborious biochemical assays [12] have shown an impaired net protein balance [13] and hypoalbuminemia [14], along with similar body composition changes [15] in co-infected individuals compared to individuals with HIV infection or TB alone. Since these techniques do not provide a comprehensive picture of the metabolic state, metabolomics approaches would greatly contribute to the existing knowledge in the field [12]. Although metabolomics has been used to better understand specific aspects of host metabolism during co-infection, such as tryptophan metabolism [16–18], an explorative approach to characterize the metabolic alterations that accompany HIV/TB co-infection has not been used. Untargeted metabolomics aims to characterize the metabolome holistically by identifying and quantifying as many metabolites as possible in a sample. It is also considered a hypothesis-generating research approach [19] which guides more focused or targeted research later. Therefore, untargeted metabolomics is ideal to provide a preliminary overview of the altered host metabolism during HIV/TB co-infection. From this, possible metabolic mechanisms associated with the HIV/TB synergy can be identified which will highlight areas of interest for future studies.

The altered gut microbiome caused by HIV infection [20] and TB [21] have been reported previously, although not well described or understood, and could play a role in host metabolism [22]. The metabolic effects of an altered gut microbiome linked to HIV/TB co-infection have not yet been investigated, however, this would be considered an important research interest considering the morphological changes previously reported in the intestines of co-infected patients [23]. Given the systemic nature of HIV infection and TB, as well as the potential involvement of microbial metabolites, it is preferable to be able to detect as many metabolites as possible. For this, two-dimensional gas chromatography time-of-flight mass spectrometry (GCxGC-TOFMS) is ideal. The advantages of this technique include increased peak capacity, resolution, and sensitivity [24].

While antiretroviral therapy (ART) typically leads to decreased viral load, low-level inflammation persists, and metabolic complications are common in PLWHA [25]. The metabolic effects of ART in the context of HIV/TB co-infection has been investigated using conventional techniques (reviewed by Jain et al. [25]), and with a focus on the immune reconstitution inflammatory syndrome, using metabolomics [26]. Jain et al. [25] indicated that the metabolic complications experienced by patients vary even among those taking different antiretroviral drugs from the same class. The authors attributed this varied response to environmental and genetic factors, as well as duration of infection. However, to date, metabolomics has not been used to compare untreated and treated HIV/TB co-infection.

In this investigation, serum samples were collected from healthy controls, as well as patients with TB, and HIV/TB co-infection, with and without ART (all these individuals were untreated for TB). Since the sample groups were small, non-homogenous (in terms of the extent of disease and duration of ART) and lacking in associated clinical indicators (such as viral load in most cases), the statistical power would be too low to identify specific metabolites that could be investigated as accurate markers of, or therapeutic targets for, the HIV/TB co-infected state. Hence, metabolomics was used in a solely explorative manner as a first step for future studies. The aim was to identify areas of metabolism, or classes of metabolites, that could potentially be investigated in more detail in future studies, and to get an indication of how the metabolomics results would compare to what is already known. Given the inherent complexity of HIV infection and TB, multidisciplinary approaches such as metabolomics which incorporate several disciplines to address a research question, are crucial to understanding the mechanisms behind these infections. Including metabolomics techniques would therefore be highly informative, but requires some preliminary information, such as that provided here. As metabolic information could be useful to researchers investigating the co-infected state from perspectives other than metabolomics as well, these preliminary results and the ideas generated thereby, are explored here in the context of existing literature. In a previously published review, we generated hypotheses regarding the metabolic profile of HIV/TB co-infected patients, measurable by metabolomics [12]. Here, we expand on those hypotheses using data generated from the above-mentioned samples. These samples were analyzed using two-dimensional gas chromatography time-of-flight mass spectrometry to get as wide a view of the metabolism as possible.

## Methods

### Study participants

Serum samples were collected by the South African Tuberculosis Vaccine Initiative (SATVI) and the Desmond Tutu HIV Centre at the Institute of Infectious Diseases and Molecular Medicine, University of Cape Town, according to standard protocols [27] and cryopreserved at -80°C. For this retrospective exploratory study, de-identified samples were transported on dry ice to the North-West University, Human Metabolomics: Laboratory of Infectious and Acquired Diseases, where it was immediately placed in a -80°C freezer until the day of analysis.

All study participants were South African adults (male and female, between the ages of 18 and 69), residing in the Masiphumelele and Ocean View Townships and surrounding areas, in Cape Town, in the Western Cape Province of South Africa. Samples were collected upon first diagnosis or a returning clinic visit. As this is a retrospective study, information regarding timing of sampling in relation to duration of infection (of both HIV and *Mtb*) and of active disease (of TB) is limited to what was included in the reports from the clinic. The samples were divided into groups based on serum-confirmed HIV and TB status (pulmonary; diagnosed using GeneXpert), and HIV treatment status. All patients were untreated for TB; hence sampling was done at time of diagnosis. For HIV testing, consent was obtained, and pre- and post-test counseling was provided by trained staff. If a volunteer did not wish to know their HIV status result, they were excluded prior to testing. Two rapid HIV antibody tests, from different companies, was performed by trained staff, in accordance with South African/World Health Organization (WHO) guidelines, which state that two positive rapid tests constitute a diagnosis of HIV infection. Any positive HIV result was followed by a referral to appropriate health care services for further management. No anonymous, post-hoc HIV test was ever performed on collected samples. Regarding the ART regimens, patients received a fixed-dose combination of two nucleoside reverse transcriptase inhibitors (NRTIs; Tenofovir and Emtricitabine) and one non-nucleoside reverse transcriptase inhibitor (Efavirenz), except for two individuals who received two NRTIs (Abacavir with Lamivudine and Combivir, respectively) and two protease inhibitors (Lopinavir and Ritonavir).

### Study design

The metabolic profiles of 29 healthy controls (HIV-/TB-/Tn-), 22 untreated HIV-negative TB-positive individuals (HIV-/TB + /Tn-), 9 untreated HIV/TB co-infected individuals (HIV + /TB + /Tn-) and 12 HIV/TB co-infected individuals on ART (HIV + /TB + /Tn +) were measured. Following sample exclusions (based on findings under the Results section), these numbers were reduced to 24 HIV-/TB-/Tn-, 22 HIV-/TB + /Tn-, 7 HIV + /TB + /Tn- and 12 HIV + /TB + /Tn + participants. Obtaining untreated HIV, TB and co-infected samples is extremely difficult in light of the “Test and Treat” policy for HIV infection and TB, as recommended by the WHO [28, 29]. However, studies on untreated samples aid in distinguishing the effects of the infection from the effect of treatment. This is necessary if the results of future studies are to be used for the development of medical interventions, especially for the prognosis and management of those already infected, as opposed to the development of diagnostic approaches. Therefore, this pilot study was conducted using available untreated and treated samples to inform and optimize future study design. Given the background of the study participants, and their limited access to and/or use of healthcare services, the exclusion criteria were not too extensive to ensure adequate sample numbers. Individuals were excluded from the study if they: (a) had another disease similar to those under investigation (e.g., asthma); or (b) were pregnant or lactating. Given that the burden of both HIV/AIDS and TB is highest in resource-limited areas [3, 30], these exclusion criteria increase the robustness of the results.

### Metabolomics analyses and data processing

A quality control (QC) sample was created by combining 10 µL of each sample and using one 50 µL aliquot of this pooled QC sample per sample batch. Extraction and analysis of the patient and QC samples were done as previously described [31]. Briefly, 50 µL of serum was extracted using a methanol:chloroform:water (1:3:1) solution (containing 50 parts per million 3-phenylbutyric acid as internal standard). Proteins were precipitated using acetonitrile. Extracts were dried with nitrogen gas, followed by derivatization using methoxyamine and N,O-bis(trimethylsilyl)trifluoroacetamide with 1% trimethylsilyl chloride. The derivatized samples were placed into the sample tray of a Gerstel Multi-Purpose Sampler (Gerstel GmbH and co.KG, Eberhard-Gerstel-Platz 1, D-4573 Mülheim an der Ruhr, Germany) coupled to a two-dimensional gas chromatographer time-of-flight mass spectrometer (GCxGC-TOFMS; Pegasus 4D; LECO Corporation, St. Joseph, MI, USA). The GCxGC-TOFMS had an Agilent 7890A gas chromatograph (Agilent, Atlanta, GA) and a TOFMS from LECO Corporation, equipped with a cryogenic cooler. All patient and QC samples were injected (1 µL) with a 1:5 split ratio, at an inlet temperature of 270°C (which remained constant throughout the run). The first four injections per batch included one injection of a mixture of fatty acyl methyl esters (FAMEs) and three QC sample injections (to prime the analytical apparatus). The following sequence was then repeated for all sample batches, until all the samples in a batch had been injected: an injection of hexane (to avoid carry-over), a QC injection, and 5–6 randomized patient samples. After the last patient sample had been injected, the run ended with a hexane injection, three QC injections, and finally a FAMEs injection. A Restek Rxi-5Sil-MS primary capillary column (28.75m, 0.25 mm internal diameter and 0.25 µm film thickness) and a Restek Rxi-17 secondary capillary column (1.38m, 0.25 mm internal diameter and 0.25 µm film thickness) were used to achieve compound separation. The oven, modulator and mass spectrometer programming were done as previously described [31], except for hot and cold pulses of nitrogen gas every 3 s for 1.5 s.

The data generated was processed using ChromaTOF software version 4.32 (LECO Corporation). Peak detection and deconvolution were performed before aligning all samples. The National Institute of Standards and Technology [32] mass spectral libraries and that of the Potchefstroom Laboratory for Inborn Errors of Metabolism were searched to identify peaks. The dataset was normalized using mass spectral total useful signal [33] to reduce technical variation and make samples more comparable. The normalized dataset was then subjected to various clean-up steps, including a 50% zero filter [34], batch effect correction using quantile equating [35] and a QC coefficient of variation (CV) filter [36].

### Statistical methods

#### Strategy and rationale

**Fig. 1:**
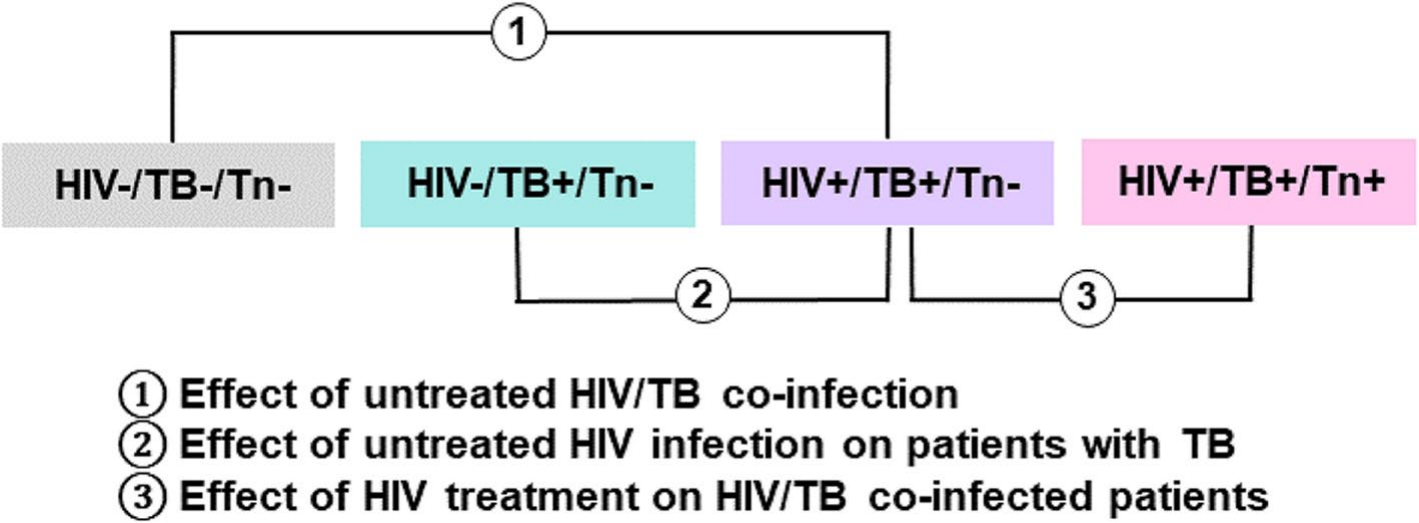
Comparisons pertinent to the aims of this investigation include; Aim 1: Determine the effect of untreated HIV/TB co-infection on the host metabolism by comparing a healthy control group to an untreated co-infected group; Aim 2: Determine the effect of untreated HIV infection on patients with TB by comparing an untreated co-infected group to an untreated group with TB only (no HIV infection); Aim 3: Determine the effect of ART on HIV/TB co-infected patients by comparing an untreated co-infected group to a co-infected group receiving ART. Groups: HIV-/TB-/Tn-: healthy controls; HIV-/TB + /Tn-: untreated HIV-negative patients with active pulmonary TB; HIV + /TB + /Tn-: untreated HIV/TB co-infected patients; HIV + /TB + /Tn + : HIV/TB co-infected patients on ART

Since this study is solely explorative, a wide array of statistical tests was applied to expand the pool of potentially important metabolites, from which to generate hypotheses. The groups were compared univariately in the following manner: (i) compare all the untreated patient samples as one group to the healthy controls using a t-test and fold change (FC) to identify metabolic changes common to all the infection/diseased states in the absence of ART; (ii) compare all groups using one-way analysis of variance (ANOVA) to identify metabolic changes unique to each infected/disease state. In the ANOVA, only those comparisons pertinent to this investigation were explored (Fig. 1). Similarly, all the comparisons listed above were also subjected to principal component analysis (PCA), partial least squares-discriminant analysis (PLS-DA) and hierarchical clustering analysis (HCA) for multivariate data analysis exploration. The decision to show either PCA or HCA here, for visualization, was made based on which of the two analyses were more informative for that specific comparison. Furthermore, the untreated co-infected samples were stratified based on CD4 T-cell count into groups with lower CD4 T-cell count (LCD; < 1–100 cells/mm^3^) and higher CD4 T-cell count (HCD; 101–650 cells/mm^3^) to observe metabolic extremes when CD4 T-cell count serves as a measure of disease severity. The point of division at 100 cells/mm^3^ was the natural point of separation in the group, as approximately half of the group had CD4 T-cell counts below 100 cells/mm^3^ (four of nine samples; 44.4%), while the other half had counts above 100 cells/mm^3^ (five of nine samples; 55.6%). The LCD and HCD groups were then compared using t-test, FC, and HCA. Analysis of the role of CD4 in the treated co-infected group was considered, but not done, due to the large heterogeneity displayed by this group (Table S1).

#### Statistical analysis

The distributions of nominal variables were assessed using Fisher’s exact tests in IBM SPSS Statistics 27 software. Missing value replacement, data pre-treatment, and statistical analysis were performed on MetaboAnalyst (version 5.0). As per default, missing values were replaced by one-fifth of the minimum value in the dataset for each variable and is based on the hypothesis that these missing values are likely not truly zero but the result of compound abundances below the detection limit of the instrument [37]. The datasets were transformed and scaled (the specific method applied depended on the dataset) to achieve a normalized distribution, as this is a prerequisite for the accurate usage of various statistical analyses. Univariate analyses included t-tests, FC, and ANOVA with correction for multiple testing by the false discovery rate (FDR) approach (variables were considered statistically significant if the adjusted *p*-value was < 0.05) and Fisher’s least significant difference for post-hoc analysis.

Only biologically relevant metabolites were subjected to Pearson’s r correlations due to the size of the dataset. Correlations were only considered significant if the adjusted *p*-value was < 0.005, to further reduce the number of metabolite markers to a usable amount for data interpretation and increase the confidence in the results.

As mentioned, multivariate analyses included PCA (used to assess the inherent structure of the data) and PLS-DA. PLS-DA models were subjected to leave-one-out cross-validation and permutation testing (precision accuracy during training) but was not used to identify predictive markers. Instead, PLS-DA variable influence on projection (VIP) values, which directly correlate with the importance of a metabolite in differentiating the groups [38], were used in an explorative sense. That is, to assess the distribution of groups and determine whether the metabolites identified as statistically significant in the univariate analyses retained significance in a multivariate statistical setting.

## Results

A total of 529 compounds were detected in this study before data clean-up. Several statistically significant metabolites could not be correctly annotated, possibly due to low signal-to-noise ratios, unsuccessful deconvolution, or its absence from the libraries used [39]. Metabolites that could not be related to any biological function were labelled as “exogenous/unannotated metabolites” and are not discussed, thus their presence and function in human serum requires further investigation. Instances where metabolites were annotated with the same name but with different unique masses and where the mass spectra could not be visually matched, were listed separately, with the unique mass as identifier.

### Clinical and demographic parameters of study participants

The number of samples per group, as well as demographic and clinical information of the cohort is given in Table 1. See Table S1 for detailed information on PLWHA. CD4 T-cell counts were not recorded for HIV-negative TB-positive individuals as part of clinical practice, and hence this information was not available. Two cases within the untreated co-infected group presented with very high CD4 T-cell counts. Table 1 shows the mean CD4 T-cell counts when these two cases are included and excluded (below the double-line). Note that additional samples were also excluded from the healthy group, as discussed later in the Results Section.

**Table 1:** Cohort demographics and clinical information

	**HIV-/TB-/Tn-**	**HIV-/TB + /Tn-**	**HIV + /TB + /Tn-**	**HIV + /TB + /Tn + **
No. of patients (%)	29 (40.3)	22 (30.6)	9 (12.5)	12 (16.7)
Age (years), mean ± SD (range)	33.4 ± 10.3	34.4 ± 12.8	31.9 ± 6.2	40.9 ± 10.6
Sex, male: female ratio (%)	13:16 (45:55)	13:9 (59:41)	5:4 (56:44)	4:8 (33:67)
Smokers in the group (%)	9 (31.0)	11 (50.0)	2 (22.2)	3 (25.0)
CD4 T-cell count (cells/mm^3^ blood), mean ± SD, before exclusion	N/A	N/A	221.4 ± 252.4	182.7 ± 162.2
Viral load (copies/mm^3^), range ^a^	N/A	N/A	< 20–124	< detectable–513 509
Duration of ART (years), mean ± SD	N/A	N/A	N/A	4.3 ± 3.6
Patients on FDC ART combination (%)	N/A	N/A	N/A	10 (83.3)
Patients on different ART combinations (%)	N/A	N/A	N/A	2 (16.7)
No. of patients (%), after exclusion	24 (36.9)	22 (33.8)	7 (10.8)	12 (18.5)
Age (years), mean ± SD (range)	34.0 ± 10.3	34.4 ± 12.8	32.3 ± 5.1	40.9 ± 10.6
Sex, male: female ratio (%)	11:13 (54:46)	13:9 (59:41)	4:3 (57:43)	4:8 (33:67)
Smokers in the group (%)	8 (33.3)	11 (50.0)	2 (28.5)	3 (25.0)
CD4 T-cell count (cells/mm^3^ blood), mean ± SD, after exclusion	N/A	N/A	78.29 ± 87.44	182.7 ± 162.2

*ART* antiretroviral therapy, *SD* standard deviation, *FDC* fixed-dose combination

Groups: HIV-/TB-/Tn-: healthy controls; HIV-/TB + /Tn-: untreated HIV-negative patients with active pulmonary TB; HIV + /TB + /Tn-: untreated HIV/TB co-infected patients; HIV + /TB + /Tn + : HIV/TB co-infected patients on ART

^a^ Viral load was not available for all patients, hence the data shown here are not representative of the entire group. CD4 T-cell counts, and viral load also varied due to differences in ART duration

**Fig. 2:**
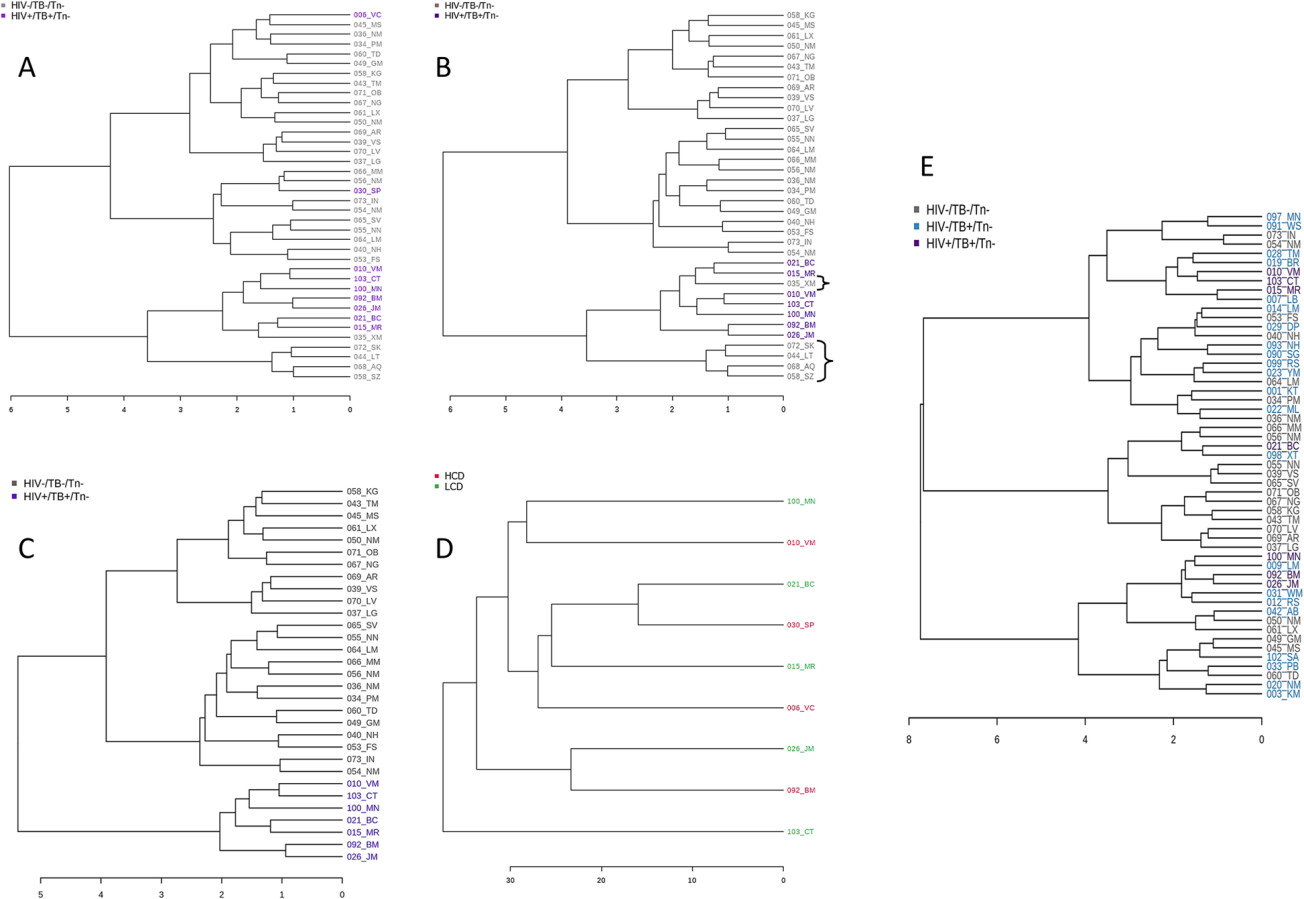
Pearson’s dendrograms (**A**) of the healthy control and untreated HIV/TB co-infected samples (*n* = 38); (**B**) after the exclusion of untreated HIV/TB co-infected samples with CD4 T-cell counts above 600 cells/mm^3^ (*n* = 36); (**C**) after the exclusion of five healthy controls (*n* = 31); (**D**) of the untreated HIV/TB co-infected samples after stratification based on CD4 T-cell count before sample exclusion (LCD: lower CD4 count, < 100 cells/mm^3^, HCD: higher CD4 count, > 100 cells/mm^3^, *n* = 9); and (**E**) the healthy control, untreated TB-positive and untreated HIV/TB co-infected samples after the exclusion of the seven samples (*n* = 53). The distance measure used in these dendrograms was Pearson’s and the clustering algorithm, Ward’s D

Two samples in the untreated co-infected group with CD4 T-cell counts above 600 cells/mm^3^ clustered with the healthy controls during hierarchical clustering (Fig. 2A), despite not being flagged as outliers. Samples were considered outliers if the average of all observations for that sample lay more than 1.96 standard deviations above or below the mean (none of the samples were outliers based on this). This implied that these participants had not yet experienced significant metabolic aberration due to the dual infection. This may be due to a host of factors including, but not limited to, the duration of their HIV infection or a genetic advantage that confers protection during HIV infection (such as specific configurations of the *CCR5* and *HLA* class I loci [40]). As such, HCA was repeated excluding the two samples with high CD4 T-cell counts. The mean CD4 T-cell count after exclusion was 78 ± 87 cells/mm^3^, which is suggestive of a more severe or more progressive diseased state. As expected, exclusion of these samples resulted in a tighter clustering of the samples within a group and greater separation between groups in the hierarchical clustering dendrogram, except for five healthy control samples, which clustered within the region of the co-infected samples (marked with brackets, Fig. 2B). Although there were no declared clinical factors that could have resulted in this clustering, it is possible that these participants had pathologies unknown to themselves or if known, they simply did not declare them. Additionally, there were no significant demographic differences between the compared groups for age, sex, smoking, and CD4 T-cell counts (in PLWHA) using Fisher’s exact test (Table S2). This dendrogram clustering suggests a possible role for metabolomics in the detection of conditions with similar metabolic profiles to that of HIV/TB co-infection. These five samples were excluded to ensure as homogenous a healthy control group as possible to increase the confidence in the results (Fig. 2C), resulting in *n* = 24 for the healthy control group, as reflected in Table 1. When the untreated patient cases, i.e., TB-positive and co-infected groups, were subjected to HCA after the sample exclusions, the samples from the respective groups mainly overlapped (Fig. 2E). This implies that there are determinants other than, or in addition to, disease status and CD4 T-cell count that drive this clustering. See Tables S3–S4 and Fig. S1 for more detail about the stratification and exclusion of samples.

Corroborating this trend, when the untreated HIV/TB co-infected samples were stratified based on CD4 T-cell count, before the exclusion of samples with high CD4 T-cell counts (Fig. 2D), it was clear that there were other sources of variation since the differences within the LCD and HCD groups were larger than those between the groups. One such factor could be viral load, which was not recorded for all participants at the time of sample collection. All further analyses were thus done excluding the five healthy controls that clustered with the co-infected samples and the two co-infected samples with high CD4 T-cell counts.

#### Influence of CD4 T-cell count on the metabolic profile of untreated co-infection

**Fig. 3:**
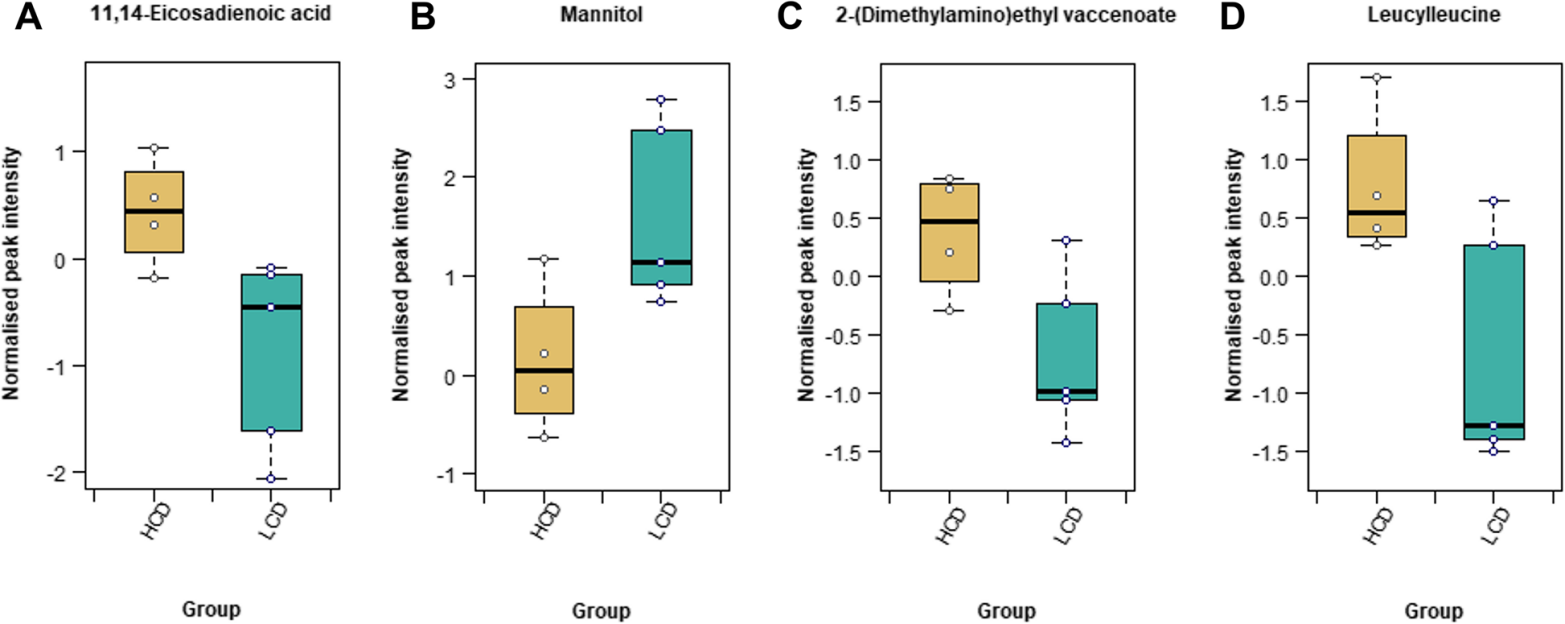
Box plots with overlaid strip plots showing the distribution of the data for the metabolites significantly altered in the t-tests comparing the HCD (*n* = 5) and LCD (*n* = 4) groups within the untreated HIV/TB co-infected group before exclusion of samples with high CD4 T-cell counts. Analysis of the role of CD4 in the treated co-infected group was not done due to the large heterogeneity displayed by this group. These results were not statistically significant after multiple testing, but as this is an explorative study, *p*-values before correction are reported here: (**A**) 0.032, (**B**) 0.037, (**C**) 0.038, and (**D**) 0.041

When the HCD and LCD groups were compared using t-tests, no metabolites were significantly different after correction for multiple testing. However, since the groups are small, the results without FDR were also explored. Six metabolites had *p*-values < 0.05 (without FDR) using t-tests and had FCs > 2: 11,14-eicosadienoic acid, mannitol, 2-(diethylamino)ethyl vaccenoate, leucylleucine, and two unannotated compounds (Tables S5 and S6). Thus, lipid and lipid-like molecules, protein catabolism, and organic oxygen compounds appear to be associated with CD4 T-cell counts as these metabolites were comparatively reduced in the group with a more severe disease state based on CD4 T-cell counts. Figure 3 shows the distribution of these compounds in the HCD and LCD groups within the untreated HIV/TB co-infected group.

### Comparing the untreated patient samples to healthy controls 

**Fig. 4:**
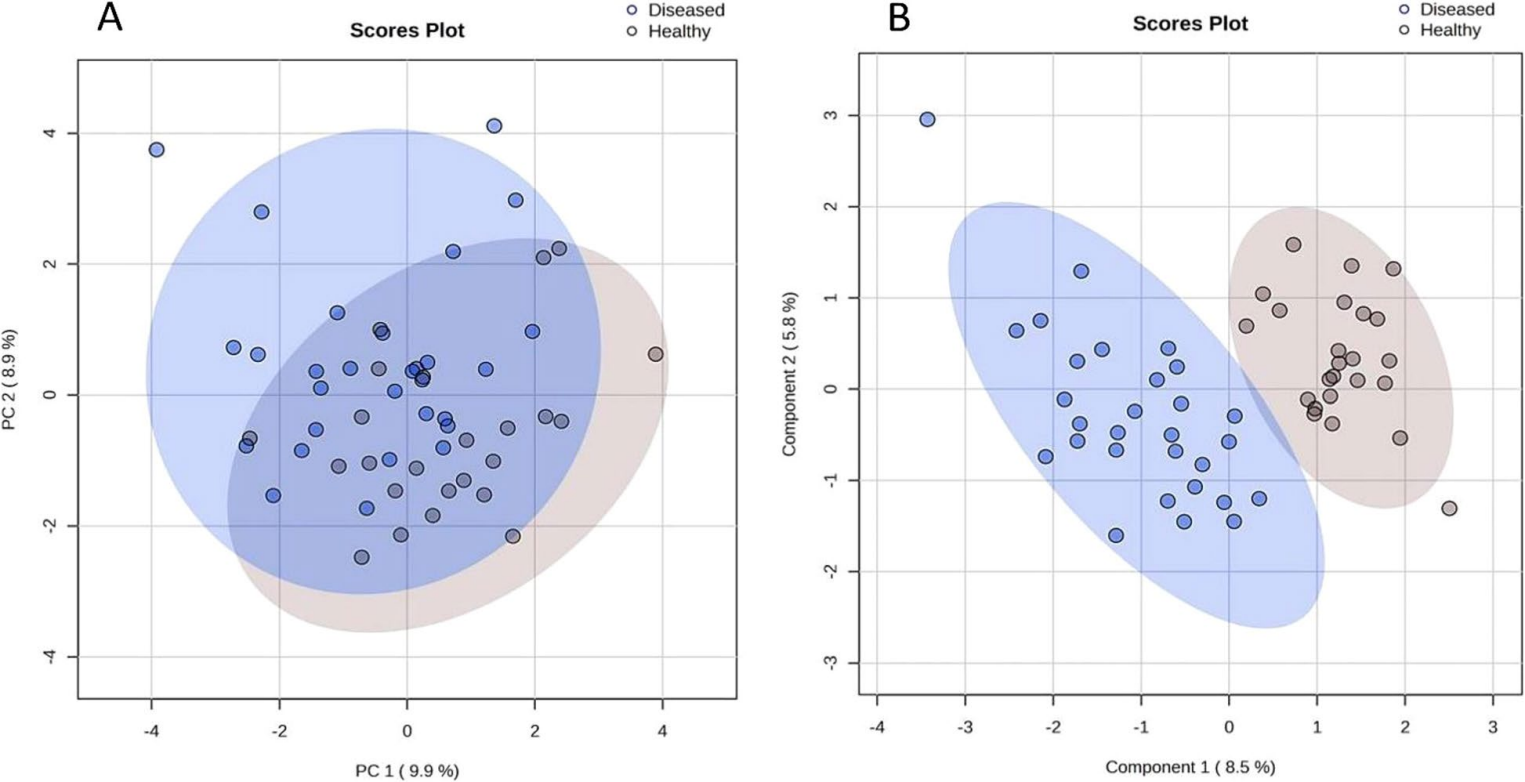
**A** PCA and (**B**) PLS-DA scores plots indicating the distribution of samples (healthy controls and all untreated patient samples, *n* = 53). Groups mainly overlapped at (**A**) but were more homogenous and could be better distinguished from each other at (**B**) mainly because of changes linked to inflammation and the breakdown of molecules as fuel during chronic inflammatory diseases such as this. While the PLS-DA model (**B**) did not perform optimally in cross-validation for a one-component model (accuracy = 0.54), it did validate during permutation testing (*p*-value =  < 0.05)

Since HIV infection and TB are known to cause overlapping metabolic changes [12], all the untreated patient samples (HIV-/TB + /Tn- and HIV + /TB + /Tn-, *n* = 29) were grouped together and compared to the healthy controls (*n* = 24) to get an indication of which metabolites are related to the general response to infection as opposed to those associated with either HIV infection or TB separately. Figure 4A is a PCA scores plot of this comparison, which indicates that the metabolic profiles of patients partly overlap with those of the healthy controls. This is not unexpected, as this is a common occurrence in the HIV and TB metabolomics literature (for example, [41] and [42]), and may also be a consequence of the heterogeneity in the patient or control samples. L-Proline, glycerol (unique mass: 103), glycerol (unique mass: 73), D-lyxose, and 2-ketobutyric acid were significantly increased while 3-indolepropionic acid and 4-hydroxyproline were significantly decreased, all more than twofold (0.5 < FC > 2), in the untreated patient group as compared to the healthy controls. These metabolites also had a PLS-DA (Fig. 4B) VIP > 1.5, while the cut-off for significance is typically VIP > 1 (see Tables S7–S10 for detailed statistical results and Fig. S2 for the PLS-DA validation results).

### Comparing all the groups simultaneously

**Fig. 5:**
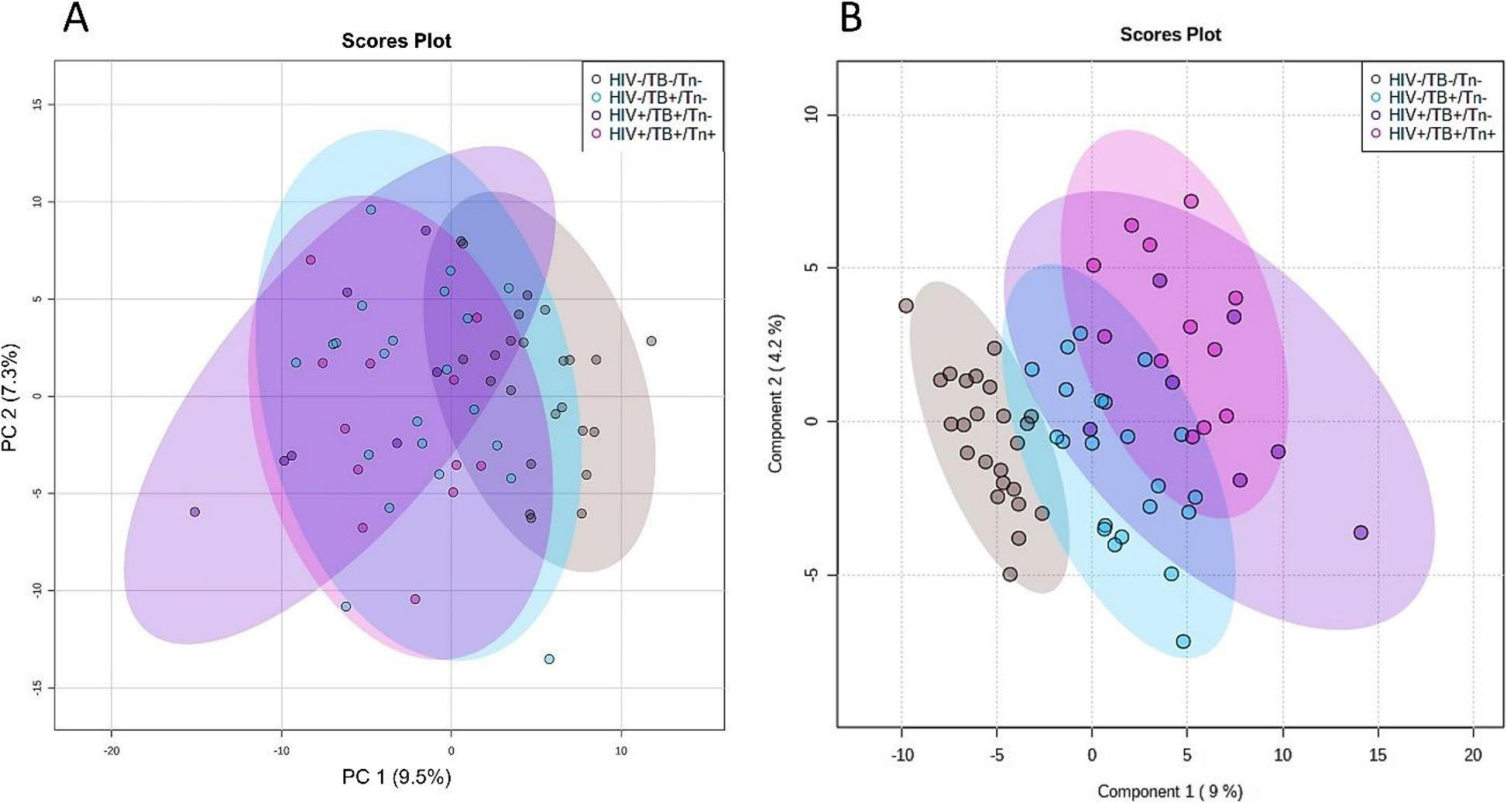
(**A**) PCA and (**B**) PLS-DA scores plots indicating the distribution of all samples (healthy controls and all untreated and treated patient samples) after sample exclusions (*n* = 65). Post sample exclusion, the groups were more homogenous and could be better distinguished from each other, more so with PLS-DA analysis and those variables important in separating the groups, identified. The PLS-DA model (B) did not validate (cross-validation for a one-component model: Q^2^ = 0.40, R^2^ = 0.60, accuracy = 0.60; permutation testing *p*-value = 0.241) and was used solely in an explorative, as opposed to predictive manner

The PCA scores plot showed no natural separation between the healthy controls and patient groups (untreated and treated), suggesting very little metabolic differences between the groups (Fig. 5A). The PLS-DA scores plot showed the patient groups (untreated and treated) mainly overlap although still more heterogeneous than the healthy controls. The healthy controls were more distinctly separated from the patient groups (Fig. 5B). The PLS-DA scores plot also indicates that the co-infected individuals on ART, and not the untreated co-infected individuals, tended to lie furthest away from the healthy controls suggesting exacerbated or distinct metabolic changes because of ART (Fig. 5B). PLS-DA cross-validation and permutation testing indicated that the model was over-fitted (cross-validation for a one-component model: Q^2^ = 0.40, R^2^ = 0.60, accuracy = 0.60; permutation testing *p*-value = 0.241), and as such, the PLS-DA model was only used in an explorative sense to identify those metabolites contributing to group separation.

**Fig. 6:**
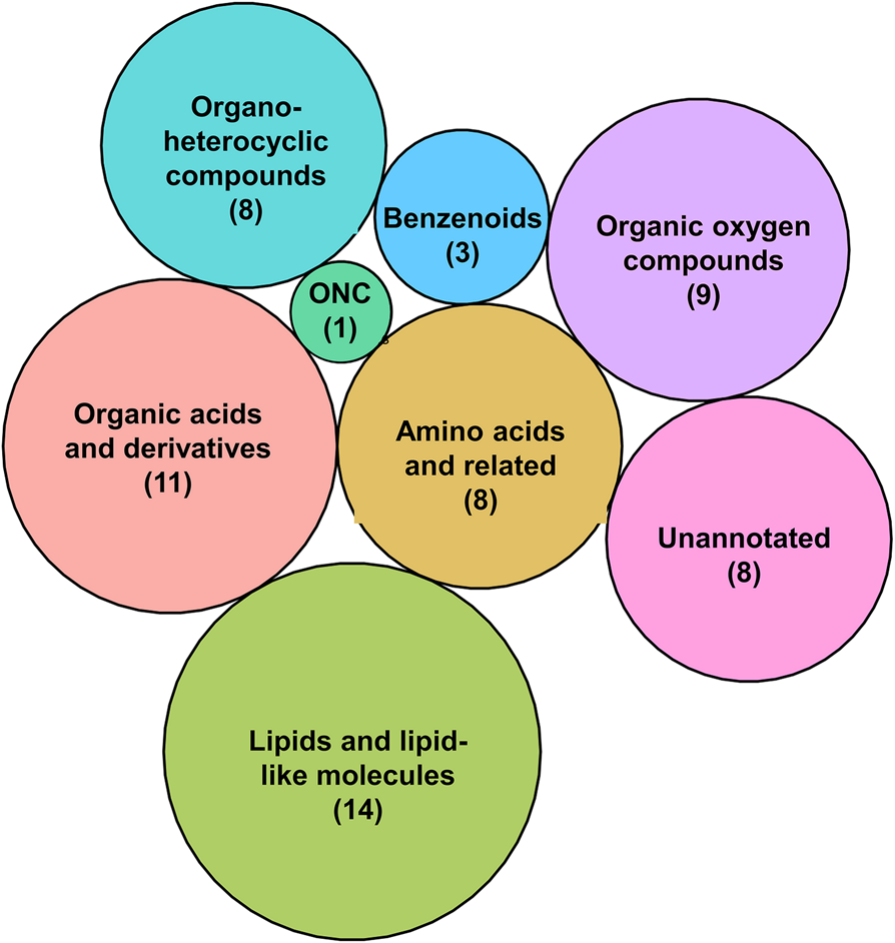
A circle packing plot showing the distribution of compound classes within the metabolites identified as significant by ANOVA. The radius of the circle is proportional to the number of metabolites of that specific class (also indicated in brackets). The plot shows lipid-like molecules as well as organic acid intermediates to be key contributors explaining the variance between the groups in this cohort. Contributing the least was the organic nitrogen compounds (ONC)

Figure 6, a circle packing plot (created in R using ggplot2 and the ‘packcircles’ package), illustrates the 62 metabolites identified as significantly different between all the groups based on ANOVA. This included various compound classes, namely lipids and lipid-like molecules, organic acids and their derivatives, amino acids, organoheterocyclic compounds, organic oxygen compounds, benzenoids and organic nitrogen compounds (also see Tables S11-13). The radius of each circle is proportional to the number of compounds in that specific class. Classes are listed as described in the Human Metabolome Database. When excluding the unannotated compounds (*n* = 8; see Table S11), this yielded 54 interpretable metabolites of which the trends and the corresponding statistics are shown in Table 2 (also see Table S13 for line graphs of all these metabolites). All metabolites indicated as significant via ANOVA also had a VIP > 1, based on the first component of the PLS-DA (as indicated by cross-validation testing, see Figure S3). Although the metabolites listed in Table 2 are not predictive, they can be used to explain the variance between the groups in this cohort, and therefore, the metabolic alterations induced during the infection/disease states under investigation. For subsequent results, of those significant metabolites identified, only the top 15% are listed in the text in order of increasing ANOVA *p*-values, in cases where the full list exceeds ten metabolites. The full lists for each comparison are given in Tables S14–S16 and the Pearson’s r correlation data in Table S17.

**Table 2:** The serum metabolites (arranged by compound class) and the associated descriptive statistics that best describe the variation between the groups compared in this study

Compound	Mean peak intensity (standard deviation)				ANOVA *p*-value (FDR)
	**HIV-/TB-/Tn- (*n* = 24)**	**HIV-/TB + /Tn- (*n* = 22)**	**HIV + /TB + /Tn- (*n* = 7)**	**HIV + /TB + /Tn + (*n* = 12)**	
**Lipids and lipid-like molecules**					
Glycerol-3-phosphate	0.653 (0.588)	-0.091 (0.79)	-1.063 (1.4)	-0.52 (0.934)	0.001
Alpha-Linolenic acid	0.597 (0.901)	-0.215 (0.59)	-1.257 (1.2)	-0.067 (0.89)	0.001
3-Hydroxyisovaleric acid	0.711 (0.672)	-0.36 (1.094)	-0.669 (0.998)	-0.372 (0.497)	0.002
9,12-Octadecadien-1-ol, (Z,Z)-/Linoleyl alcohol	-0.57 (0.742)	0.075 (0.953)	1.059 (0.906)	0.384 (0.943)	0.005
1,E-11,Z-13-Octadecatriene	0.653 (0.905)	-0.242 (0.817)	-0.516 (0.702)	-0.561 (0.999)	0.005
Glycerol monostearate	0.526 (0.609)	-0.009 (0.703)	-0.735 (2.037)	-0.606 (0.698)	0.014
1-Monopalmitin	0.6 (0.499)	-0.23 (0.86)	-0.672 (1.318)	-0.386 (1.264)	0.014
Octanoic acid	-0.473 (0.64)	0.111 (0.917)	1.086 (1.383)	0.109 (1.019)	0.021
Hexadecanoic acid/Palmitic acid	0.602 (0.463)	-0.345 (1.287)	-0.233 (0.839)	-0.435 (0.745)	0.022
Nonanamide (amide of nonanoic acid)	0.406 (0.737)	-0.044 (0.571)	-1.177 (2.215)	-0.045 (0.366)	0.022
N-(2-hydroxyethyl)-Decanamide	0.537 (0.816)	-0.143 (0.972)	-0.834 (0.946)	-0.326 (0.951)	0.023
Arachidonic acid	0.536 (0.792)	-0.303 (1.055)	-0.746 (1.367)	-0.082 (0.435)	0.030
Eicosapentaenoic acid	0.429 (0.834)	-0.01 (0.859)	-1.007 (1.011)	-0.252 (1.132)	0.041
E,E-1,9,17-Docasatriene	0.524 (0.861)	-0.483 (0.991)	-0.206 (0.916)	-0.042 (0.917)	0.043
**Organic acids and derivatives**					
3,4-Dihydroxybutyric acid	-0.877 (0.412)	0.291 (0.789)	1.206 (1.159)	0.517 (0.693)	< 0.0001
2-Propenoic acid, 2-(dimethylamino)ethyl ester/Dimethylaminoethyl acrylate	0.715 (0.805)	-0.325 (0.882)	-0.63 (0.946)	-0.467 (0.822)	0.001
Cyclobutanecarboxylic acid, 2-dimethylaminoethyl ester	0.622 (0.65)	-0.25 (1.024)	-0.843 (0.89)	-0.295 (0.992)	0.007
2,4-Dihydroxybutyric acid	0.092 (0.407)	0.464 (0.772)	-0.259 (0.482)	-0.882 (1.694)	0.015
Carbonic acid, 2-dimethylaminoethyl propyl ester	0.299 (0.529)	0.201 (0.609)	-1.216 (2.01)	-0.258 (1.015)	0.019
Pyruvate	0.549 (0.68)	-0.161 (1.063)	-0.698 (1.18)	-0.397 (0.862)	0.025
2-Hydroxyethyl palmitate	0.548 (0.699)	-0.241 (1.043)	-0.762 (1.249)	-0.211 (0.824)	0.029
1,2-Butanediol	-0.544 (0.851)	0.488 (1.012)	0.07 (0.608)	0.152 (0.998)	0.032
Hydracrylic acid/Hydroxypropionic acid	-0.489 (0.862)	0.032 (0.779)	0.678 (1.427)	0.525 (0.938)	0.039
Carbonic acid, 2-dimethylaminoethyl ethyl ester	-0.26 (0.736)	0.151 (1.186)	1.08 (1.067)	-0.388 (0.53)	0.042
2-Aminobutanoic acid	0.48 (0.814)	-0.04 (0.838)	-0.479 (1.071)	-0.609 (1.186)	0.050
**Amino acids**					
DL-Phenylalanine	-0.774 (0.594)	0.317 (0.882)	0.927 (0.72)	0.427 (1.028)	< 0.001
L-Lysine	0.592 (0.649)	-0.003 (0.635)	-1.065 (1.408)	-0.556 (1.125)	0.001
L-Proline	0.633 (0.644)	-0.115 (0.785)	-0.466 (0.534)	-0.784 (1.382)	0.003
Phenylalanine	0.667 (0.656)	-0.352 (1.035)	-0.784 (1.144)	-0.232 (0.701)	0.004
L-Hydroxyproline	-0.577 (0.653)	0.131 (1.047)	1.093 (0.72)	0.275 (0.974)	0.004
L-Tryptophan	0.585 (0.88)	-0.363 (0.926)	-0.82 (1.177)	-0.027 (0.606)	0.011
L-Threonine	0.508 (0.557)	-0.047 (0.752)	-1.021 (1.608)	-0.333 (1.153)	0.013
L-Methionine	0.574 (0.836)	-0.266 (0.937)	-0.802 (0.448)	-0.193 (1.123)	0.015
**Organoheterocyclic compounds**					
Tetrahydrofuran	0.797 (0.49)	-0.13 (0.588)	-1.057 (0.915)	-0.739 (1.212)	< 0.0001
1,2,4,5-Tetrazine-3,6-diamin	0.273 (0.556)	0.39 (0.617)	-0.352 (0.997)	-1.055 (1.463)	0.003
D-Erythro-Pentofuranose	0.588 (0.705)	-0.132 (0.951)	-0.84 (1.287)	-0.445 (0.832)	0.010
Indole-3-lactic acid	-0.607 (0.981)	0.445 (1.043)	-0.07 (0.379)	0.44 (0.484)	0.011
1H-Indole-3-acetic acid	0.564 (0.761)	-0.205 (1.111)	-0.674 (0.951)	-0.358 (0.74)	0.026
D-Rhamnose	0.209 (0.526)	0.288 (0.511)	-1.154 (1.995)	-0.273 (1.175)	0.030
2-Amino-1-methyl-1H-imidazol-4-ol	0.49 (0.777)	-0.288 (0.869)	-0.786 (1.505)	0.007 (0.893)	0.043
3-Indolepropionic acid	0.364 (0.99)	0.038 (0.914)	-1.058 (0.798)	-0.182 (0.887)	0.050
**Organic oxygen compounds**					
1-Deoxypentitol	-0.499 (0.805)	-0.255 (0.78)	1.133 (1.181)	0.805 (0.584)	< 0.001
Glucuronic acid	-0.65 (0.764)	0.296 (0.937)	0.996 (1.02)	0.176 (0.769)	0.002
Glycerol (103)	-0.492 (0.948)	0.308 (0.76)	1.171 (0.837)	-0.264 (0.888)	0.004
3,6-Dimethyl-octan-2-one	0.601 (0.468)	-0.286 (0.959)	-0.943 (1.713)	-0.126 (0.685)	0.005
Glycerol (73)	0.663 (0.748)	-0.41 (1.02)	-0.414 (0.975)	-0.333 (0.786)	0.005
D-Lyxose	-0.58 (0.921)	0.119 (0.902)	0.973 (0.818)	0.373 (0.782)	0.009
Benzoic acid	0.613 (0.717)	-0.224 (1.056)	-0.663 (0.888)	-0.428 (0.886)	0.011
Dodecanal/Lauric aldehyde	0.28 (0.489)	0.187 (0.629)	-1.266 (2.109)	-0.164 (0.919)	0.017
D-Mannitol	0.312 (0.772)	0.229 (0.952)	-0.945 (1.424)	-0.493 (0.767)	0.032
2,6-Di-tert-butyl-p-cresol	0.563 (0.828)	-0.31 (0.978)	-0.52 (1.179)	-0.255 (0.821)	0.032
**Benzenoids**					
2,4-Di-tert-butyl-phenol	0.717 (0.775)	-0.648 (0.778)	-0.47 (1.212)	0.029 (0.707)	<0.001
m-Cresol	0.412 (0.665)	-0.007 (1.078)	-1.033 (1.466)	-0.209 (0.639)	0.042
**Organic nitrogen compounds**					
Cadaverine	0.621 (0.682)	-0.367 (0.971)	-0.57 (1.238)	-0.238 (0.912)	0.011

*Abbreviations*: *HIV* human immunodeficiency virus, *TB* tuberculosis, *Tn* antiretroviral therapy, *ANOVA*: one-way analysis of variance, *FDR* false discovery rate

#### The effect of untreated HIV/TB co-infection on the serum metabolic profile

The metabolites listed in Table 2 were significantly altered in the untreated HIV/TB co-infection group relative to the healthy controls, except for glucuronic acid, D-lyxose, cadaverine, pyruvate, 2,6-di-tert-butyl-p-cresol, 2-amino-1-methyl-1H-imidazol-4-o, and an unannotated compound (see Table S14). The ten metabolites with the most significant *p*-values in this comparison, in order of significance, were 3,4-dihydroxybutyric acid (DHBA), tetrahydrofuran, phenylalanine, 1-deoxypentitol, 2,4-di-tert-butyl-phenol, glycerol-3-phosphate, alpha-linolenic acid, lysine, dimethylaminoethyl acrylate and 3-hydroxyisovaleric acid. This profile is mainly suggestive of inflammation and elevated catabolism to supply intermediates to comply with the increased energy demands, which is typical during infection [11]. It also indicates that multiple areas of metabolism are significantly affected during co-infection, as five of the seven chemical classes represented by the ANOVA results are represented in the ten most significant metabolites here.

#### The effect of HIV infection on the TB-positive serum metabolic profile

**Fig. 7:**
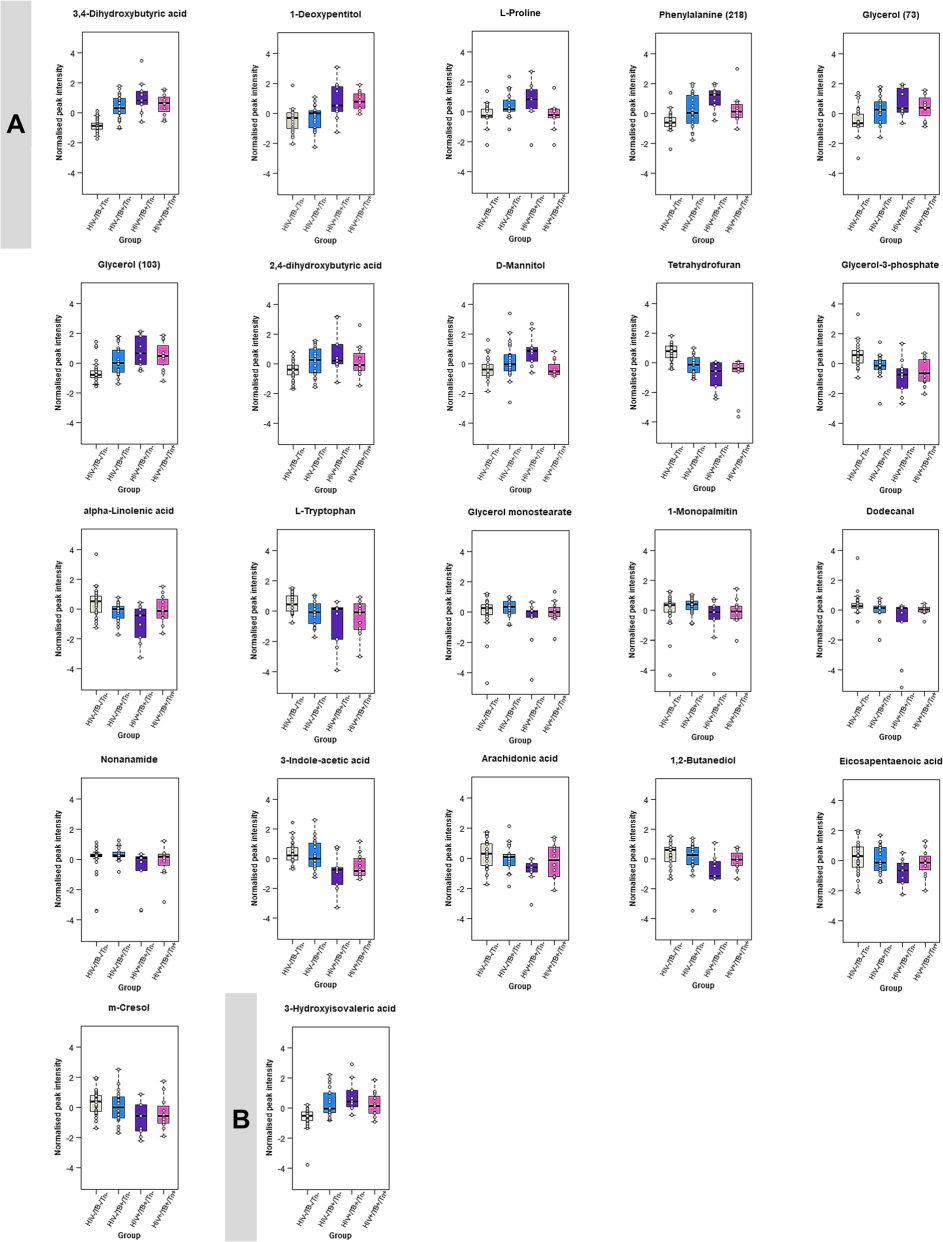
Boxplots with overlaid strip plots showing the distribution of the data for the metabolites significantly altered in the following comparisons: HIV-/TB + /Tn- (TB) versus HIV + /TB + /Tn- (untreated HIV/TB) and HIV + /TB + /Tn- (untreated HIV/TB) versus HIV + /TB + /Tn + (ART-treated HIV/TB). (**A**) Metabolites significantly altered by HIV infection during an existing TB infection, of which this trend was reversed to some extent by ART (although not with statistical significance in all cases). (**B**) 3-Hydroxyisovaleric acid was the only metabolite not significantly altered by HIV infection in those with untreated HIV/TB co-infection but was significantly altered because of ART in co-infected individuals

Figure 7A represents box plots for the metabolites that were significantly different when comparing the untreated TB-positive and co-infected samples, as well as when comparing the untreated to the treated co-infected samples. These metabolites also occurred in the untreated co-infection versus healthy controls comparison. As such, these metabolites were selected for further interpretation as they are most pertinent to the aims of this study i.e. (i) to determine the effect of untreated HIV/TB co-infection on host metabolism by comparing a healthy control group to an untreated co-infected group, (ii) to determine the effect of untreated HIV infection on patients with TB by comparing an untreated co-infected group to an untreated group with TB alone (no HIV infection), and (iii) to determine the effect of ART on HIV/TB co-infected patients by comparing an untreated co-infected group to a co-infected group receiving ART (see Fig. 1). The majority of these 13 metabolites (see Table S15) that were significantly different when comparing the untreated TB and HIV/TB co-infected groups, are involved in lipid metabolism. The remaining significant metabolites are classified as sugars and sugar alcohols, or amino acids and their derivatives. This is a typical metabolic profile for HIV infection, when considering previous literature [43–45]. In this comparison, the most significant metabolites, in order of significance, were 3,4-DHBA, tetrahydrofuran, 1-deoxypentitol, glycerol-3-phosphate and alpha-linolenic acid, previously associated with immune activation, inflammation, reduced antioxidant status and subsequent gut dysbiosis. Similarly, the class of metabolites most affected by HIV infection in individuals with TB, were lipids and lipid-like metabolites (seven metabolites out of the 22 significantly altered metabolites in this comparison, see Table S15).

#### The effect of ART on the HIV/TB co-infection serum metabolic profile

Treating HIV infection in HIV/TB co-infected patients with ART, resulted in a reversal of some of the trends observed in Sect. 3.3.2 (representing the effect of HIV infection on TB); 3,4-DHBA; L-proline; 2,4-DHBA and D-mannitol were increased in the co-infection group, and decreased when these co-infection patients received ART. Alpha-linolenic acid, glycerol monostearate, 1-monopalmitin, dodecanal, and nonanamide were decreased in HIV/TB co-infected patients relative to those with TB only, but increased after ART (Fig. 7A). Table S16 lists the most significant metabolites for this comparison. Similarly to HIV infection, lipid metabolism was most affected by ART, as five of the ten significantly altered metabolites were lipids or lipid derivatives, which is in line with previous knowledge of the metabolic effect of ART [46]. The significantly altered metabolites as affected by ART can be associated with infection-induced inflammation, gut dysbiosis and lipid metabolism. 3-Hydroxyisovaleric acid (Fig. 7B) was the only metabolite not significantly affected by HIV infection, though slightly increased in the untreated HIV/TB co-infected group relative to the TB group. This metabolite significantly decreased in the ART-treated co-infection group relative to the untreated co-infected group which may imply a potential ART-related mechanism for this reduction.

## Discussion

The metabolic profile associated with HIV/TB co-infection indicates significant overlap with TB alone, but also shows unique characteristics. These results highlight the significant amount of research that is still needed to fully characterize the human metabolome in general. The results were compared to existing literature to contextualize or explain the metabolic changes seen in this investigation.

Prior to addressing the aims of this study, a brief discussion will be provided on the metabolic changes associated with clinical and demographic parameters. In Fig. 3, metabolites related to lipid and protein metabolism (11,14-eicosadienoic acid, 2-(diethylamino)ethyl vaccenoate, and leucylleucine) were decreased, while mannitol was increased in the LCD group compared to the HCD group. This is expected, as individuals with a lower CD4 T-cell count would typically be at a more advanced stage of disease with higher viral loads. The greater severity of infection would place more strain on the host’s body as the virus uses more of the host resources for replication. Additionally, this might increase the success of *Mtb* in these individuals (see Kwan and Ernst [47] and Sharan et al. [7] and for reviews of the mechanisms by which this occurs), which would overwhelm the host’s metabolic system. Although highly plausible, this hypothesis cannot be confirmed in this study cohort since viral load and indicators of TB severity were unavailable. The decreased levels of 2-(diethylamino) ethyl vaccenoate in the LCD group implicates cholesterol metabolism as it is related to cholesterol ester cholesteryl 1-vaccenoate, which is a major constituent of lipoprotein particles. This is in accordance with previous results associating cholesterol and CD4 T-cell counts in untreated HIV infection [48] and the activation of lymphocytes in TB [49, 50]. Long-chain fatty acid metabolism is implicated by the decrease of 11,14-eicosadienoic acid in the LCD group, although not much is known about the role of this eicosadienoic acid derivative in human metabolism. It is interesting to note, the reduction of the dipeptide leucylleucine as one typically expects the opposite since protein breakdown products usually increase progressively during disease as the body starts using alternative fuel substrates to provide for the inflammation-driven elevated metabolic rate and energy demands. However, the reduction observed suggests that these individuals have already depleted their protein reservoirs, which supports the previously reported indications of an impaired net protein balance in co-infected individuals [13]. The increased levels of mannitol in the LCD group also supports this hypothesis. This increased mannitol may additionally indicate subclinical co-infections in the co-infected individuals and/or a more virulent microbiota composition [51, 52]. Gut inflammation has an impact on mannitol permeability in PLWHA [53], which could explain the increased mannitol observed in the LCD group, possibly indicating a more progressive phenotype. These metabolites, though detected in an extremely small cohort, give an indication of which metabolic analyses would be most pertinent to investigating the effect of CD4 T-cell count in individuals with HIV/TB co-infection. However, it is also clear from these and previous results that this will have to be done in combination with determining other biochemical parameters, body composition measurements, and importantly, viral load measurements, preferentially in a longitudinal study design.

Protein catabolism is a known metabolic consequence of HIV and TB infection [13, 14]. When exploring the data of the patient groups compared to the controls, the patient groups had comparatively elevated levels of 2-ketobutyric acid and L-proline and decreased levels of 4-hydroxyproline. This suggests the breakdown of proteins and amino acids for energy as these metabolites are intermediates of amino acid metabolism that feed into the tricarboxylic acid cycle. 3-Indolepropionic acid is another breakdown product of amino acids, specifically tryptophan, because of immune cell and microbial metabolism (this is explored in more detail below). Increased levels of glycerol (unique mass: 73 and 103) further indicate the use of alternative fuel substrates such as lipids, as a product of lipid hydrolysis. Furthermore, *Mtb* and other intracellular bacterial pathogens use glycerol-containing lipids as a fuel substrates [54]. For this, the bacteria synthesize their own phospholipases and rely on those of the host, such as phospholipase A2, which is released as part of the host’s inflammatory response [54, 55]. Arabinose was also significantly increased in the patient group and is an essential component of the *Mtb* cell wall [56] and has been associated with an altered gut microbiome as a result of COVID-19 [57] and other infections. Thus, the common metabolic profile of individuals with TB or HIV/TB co-infection suggests a state of low energy availability and an altered gut microbiome, possibly due to the chronic inflammatory nature of these diseases.

When investigating the data comparing the untreated HIV/TB co-infected group to that of untreated TB only (i.e., describing the effect of HIV infection on TB); phenylalanine was increased while tryptophan was reduced in the untreated co-infected group comparatively. Elevated phenylalanine has previously been reported in biofluids collected from untreated TB [36, 58–61] and untreated PLWHA [43, 62, 63]. This can be ascribed to a decreased phenylalanine hydroxylase (PAH) activity [62, 63] due to a tetrahydrobiopterin (BH_4_) deficiency [63] or compromised insulin secretion [36]. This likely occurs since the interferon-influenced BH_4_ is sensitive to oxidation, aggravated during the high levels of OS experienced during infection [9, 64] in the co-infected group [65, 66]. Furthermore, macrophages and dendritic cells produce neopterin at the expense of BH_4_, further depleting this, a mechanism confirmed by the fact that neopterin is known to be increased during both HIV infection [67] and TB [68], and is even more pronounced during HIV/TB co-infection [69]. Neopterin has also been shown to predict the development of active TB in PLWHA six months before a dramatic increase in viral load and reduction in CD4 T-cell count. Although neopterin was not detected as a metabolite marker in this study, the continued immune activation during HIV/TB co-infection [7] contributes to BH_4_ depletion via neopterin overproduction, decreasing PAH activity. Thus, the increased phenylalanine levels may be ascribed to OS and immune activation by HIV infection.

Altered tryptophan metabolism via the kynurenine pathway as a result of increased indoleamine 2,3-dioxygenase 1 activity is a well-established consequence of various inflammatory conditions, including HIV [43–45, 70–72], TB [36, 58, 68, 73, 74] and HIV/TB co-infection [16, 17]. The indole products of tryptophan catabolism by the gut microbiota can be produced endogenously in an interleukin-4 inducible manner in T-cells and dendritic cells [75]. The importance of tryptophan catabolism by the gut microbiota during co-infection is reflected in the decreased levels of tryptophan and 3-indoleacetic acid (IAA) (as well as 3-indolepropionic acid [IPA] and 3-indolelactic acid [ILA] in the untreated HIV/TB co-infected group when compared to the healthy controls). Tryptophan was decreased in all patient groups relative to healthy controls, and this was most pronounced in the untreated co-infected group although not statistically significant (Table 2). Olsson et al. [18] recently showed tryptophan levels to be lower during HIV/TB co-infection when testing the suitability of the kynurenine/tryptophan ratio as a tool for the identification of TB cases in an untreated cohort of PLWHA. IPA, IAA, and ILA all have roles as antioxidants, ameliorate inflammation [76–79], and regulate intestinal immunity by activating the aryl-hydrocarbon receptor transcription factor [75, 80]. Increased IPA has been associated with enhanced intestinal barrier integrity [81] and has mycobacterial-specific antibiotic activity [78], thus its decrease could relate to the loss of gut barrier integrity induced by HIV [82], intestinal damage known to occur in co-infected individuals [23], and possibly TB progression in PLWHA [78]. Typically, the catabolic products of tryptophan would increase as tryptophan decreases, however, the advanced disease state (as per CD4 T-cell counts in Table 1), high levels of OS [9, 64], and microbiome dysbiosis [20, 21] in the co-infected individuals may account for this observation. These data and previous reports confirm that HIV/TB co-infected individuals have a significantly increased tryptophan catabolism [16, 17] compared to individuals with either disease state alone, and suggests that the gut microbiota-derived tryptophan catabolites may be important indicators for the co-infected state. This could be used in conjunction with the kynurenine/tryptophan ratio, which has been suggested as a biomarker for co-infection [17, 18].

A general decrease in the lipid-related metabolites was observed in the untreated HIV/TB co-infected group relative to the untreated TB group, confirming previous reports of the effect of HIV on altering lipid metabolism [46]. Glycerol-3-phosphate is used for the rapid regeneration of reducing equivalents for oxidative phosphorylation [83] in the brain and skeletal muscle. The glycerol-3-phosphate shuttle also links fatty acid metabolism (which is typically dysregulated during HIV infection [84] and TB [85]) to glycolysis and oxidative phosphorylation [86], allowing fatty acids to be preferentially used for energy. Alpha-linolenic acid and arachidonic acid are essential polyunsaturated fatty acids (PUFAs) and are important structural components of cell membranes [87] and mediators of inflammation since they act as precursors to eicosanoids. Low levels of alpha-linolenic acid is associated with the failure to inactivate the virus during infection, resulting in virus proliferation and immunodeficiency [88]. PUFAs and glycerophospholipids are also important for distinguishing co-infected individuals who develop immune reconstitution inflammatory syndrome from those who do not [26]. Reduced levels of dodecanal (also called lauric aldehyde) were observed in the untreated co-infected group (Table S13), implicating decanoic acid metabolism [89] as an alternative energy substrate [31]. This fatty acid is typically produced as an intermediate of glycerophospholipid (specifically plasmalogens) metabolism and indicates changes in membrane turnover. It has previously been reported that plasmalogens are decreased during HIV infection [45, 90]. These lipids are essential to membrane structure and function, immunity, and serve as antioxidants to protect against lipid peroxidation [89]. Nonanamide and decamamide belong to the recently identified fatty amide sub-class of metabolites, which may have bioactive signaling functions [91]. Although not detected here, oleamide has been found to modulate the activity of 4-aminobutyric acid (GABA) receptors. Given the results discussed below, the effect of other fatty amides on signaling processes and metabolic enzymes requires further investigation, especially in HIV and its effect on TB in HIV/TB coinfected individuals.

The catabolism of GABA results in the production of 3,4-DHBA and 2,4-DHBA [92]. These hydroxy fatty acids were significantly increased in the untreated co-infected group relative to healthy controls — in fact, the untreated co-infected group had the highest levels compared to all other patient groups. 3,4-DHBA induces a feeling of satiety possibly due to its structural similarity to 3-hydroxybutyric acid [93], which is used as an alternative fuel substrate during fasting. These metabolites are also linked to poor overall health and nutritional status induced by an extremely low energy intake [94]. This suggests cachexia, which is in line with the overall indication of alternative energy substrate use and is characteristic of both HIV infection and TB [49, 95]. Elevated 3,4-DHBA has also been detected in the urine of unsuccessfully treated TB individuals [96], further confirming its association with a progressive phenotype. However, the exact mechanism of increased 3,4-DHBA and 2,4-DHBA concentrations have yet to be clarified. As such, 3,4-DHBA was subjected to correlation analysis with other significantly altered metabolites in this investigation to determine if this could yield new insights into the mechanisms of cachexia. 3,4-DHBA correlated positively with 2,4-DHBA, phenylalanine, 3-hydroxyisovaleric acid, and microbial metabolites (arabinose and rhamnose), while negatively correlating with tryptophan, IAA, threonine, 2-ketoisocaproic acid (another intermediate of leucine catabolism), and tetrahydrofuran. Therefore, given the mechanisms that result in the alteration of these correlating metabolites, 3,4-DHBA would increase with more pronounced inflammation and microbial translocation.

Microbiota metabolism is an essential component of human metabolism [22]. Phenylalanine and tryptophan metabolism by gut microbiota yields cresol and indole intermediates [97–99]. While previously shown to be elevated during HIV infection [62, 100, 101], a p-cresol derivative, 2,4-di-tert-butyl-phenol (which cannot be produced by humans [102]), and benzoic acid [103] were reduced in the untreated HIV/TB co-infected group relative to those with TB alone. The impaired gut mucosal integrity, microbial translocation, and microbiome dysbiosis (which is also a feature of TB) induced by HIV infection [20, 21, 53] are generally reflected in the pathomorphological changes seen in the small intestine of HIV/TB co-infected individuals. Considering this, the decreased concentrations in the p-cresol derivative and benzoic acid in this study, induced by HIV infection in TB positive individuals, may result from an altered microbiota and increased OS [23] given the antioxidant properties of these metabolites. Furthermore, 2,4-di-tert-butyl-phenol has been detected in the fecal metabolome of COVID-19 patients and significantly correlated with specific populations of COVID-19-altered gut microbes [57]. Thus, its presence in serum may be due to translocation from the intestine, because of the HIV-associated ‘leaky gut’. Similarly, cadaverine, a microbially-produced antioxidant polyamine, was detected in reduced amounts in the untreated co-infected patients relative to the TB patients. This metabolite has been detected in HIV infection [62] and *Mtb*-related metabolomics studies [37, 104]. Since the gut microbiota produces cadaverine, the reduction observed here is most likely ascribed to the altered gut health and microbiota composition associated with HIV infection and TB [105]. Cadaverine forms part of the glutathione metabolic pathway, and as such, its reduction may be associated with OS [106]. Furthermore, reduced levels of glutathione with concomitantly increased indicators of lipid peroxidation has been reported in HIV/TB co-infected patients relative to patients with HIV infection only (with and without ART) [9], which supports exacerbated OS during HIV/TB co-infection.

**Fig. 8:**
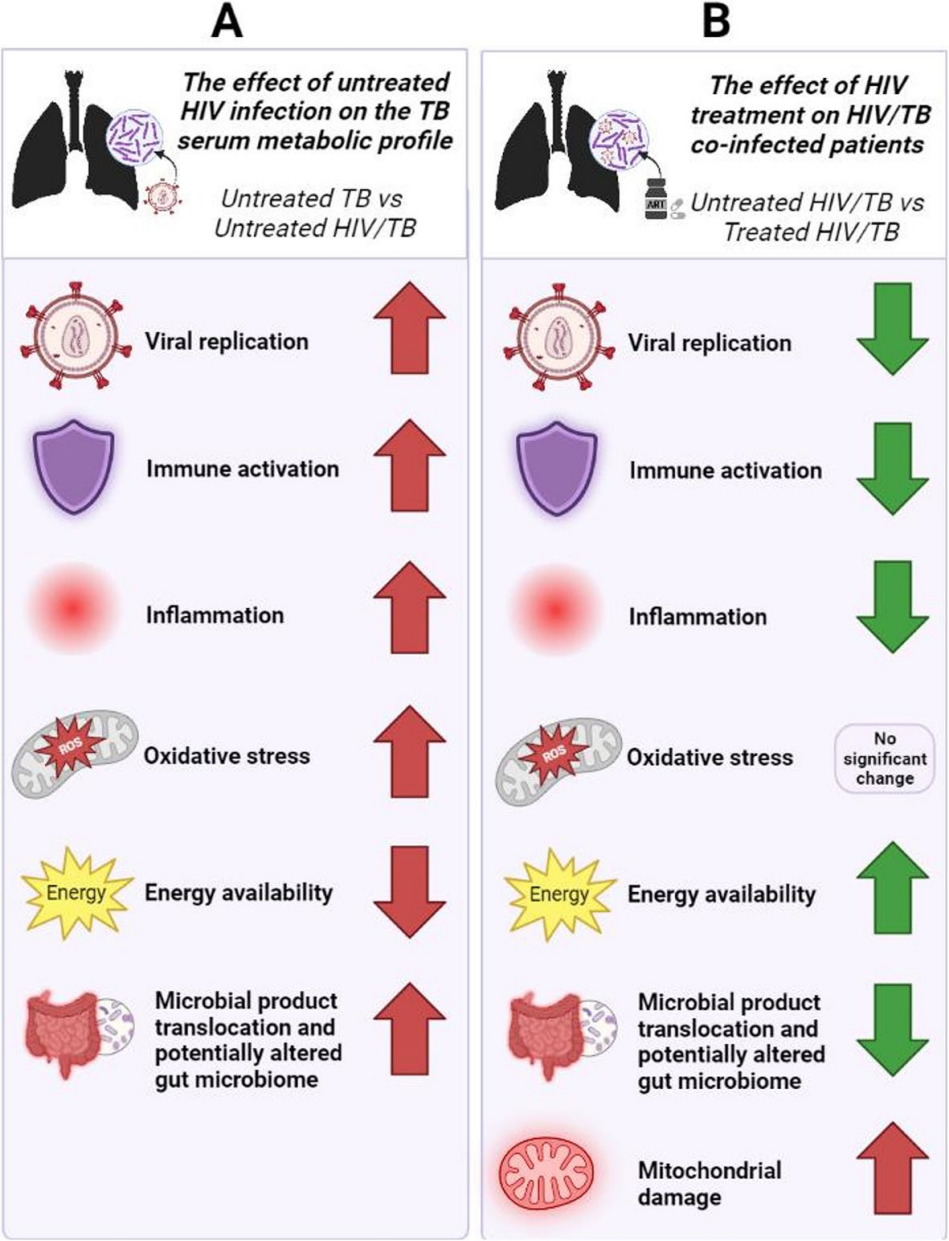
A visual summary of the potential effects of HIV infection and its treatment on patients with TB, as elucidated by this metabolomics investigation. **A** Shown are the implications of the metabolites found to be significantly different between the untreated TB and untreated HIV/TB co-infected groups, representing the effect of HIV infection on an individual who already has TB. **B** The implications of the metabolites found to be significantly different between the untreated and ART-treated HIV/TB co-infected groups, representing the effect of ART on HIV/TB co-infection. Although ART improved the effects of HIV infection to some extent, implying that some metabolites returned to a value closer to, but not equal to, those of healthy controls, many metabolites were unaffected by ART

Individuals on ART experience inflammation and immune activation, although to a lesser degree than before treatment [107]. Although ART did not generally alter the metabolic profile of the HIV/TB co-infected individuals to a statistically significant degree, the levels of alpha-linolenic acid, dodecanal and L-proline were significantly different and partially recovered (although not to the levels in healthy controls). This is in line with previous studies, which showed that ART does not restore the metabolic condition to that of healthy individuals, but rather induces further aberration [108] and still reflects continual inflammation despite viral suppression (reviewed by Hileman and Funderburg [107]). This is also reflected in the scores plot of the PLS-DA (Fig. 5B), which shows that the co-infected individuals on ART rather than the untreated co-infected individuals, lay furthest away from the healthy controls. 3-Hydroxyisovaleric acid was the only metabolite that was significantly different in co-infected individuals on ART that was not affected by HIV infection in addition to TB. Its increase may reflect the known effect of mitochondrial damage induced by ART (reviewed by Bañó et al. [109]) as it is a breakdown product of leucine. This may be a consequence of reduced methylcrotonyl-CoA carboxylase activity, as this starts a process that results in the release of the free 3-hydroxyisovaleric acid [110] but must be further investigated in this context. Figure 8 summarizes the potential effects of HIV and its treatment on HIV/TB co-infected individuals as discussed above.

## Conclusions and recommendations for future studies

HIV/TB co-infection results in a metabolic signature characteristic of what is typically seen in other infections i.e., elevated catabolism to meet the increased energy demand to fight infection. When the metabolic differences between comparative groups are analyzed more closely, the results indicate that HIV and its treatment augments TB synergistically, at least in part, through increased inflammation, OS, ART-induced mitochondrial damage, and its detrimental effects on gut health. These factors negatively impact energy availability, which weakens the host’s defenses and contributes to the increased frequency of poor outcomes for individuals with HIV/TB co-infection as opposed to those with TB alone. Although ART is typically associated with metabolic aberrations, these results indicate that it does reverse the trends caused by HIV infection for some metabolites. However, this reversal is not complete as it does not restore the metabolite levels of ART treated-PLWHA to that of healthy controls, and several other metabolites remained unchanged.

Considering the above, this investigation not only describes a metabolic signature of HIV/TB coinfection, but also gives clues to the disease mechanisms associated with HIV, TB, and the HIV/TB co-infected state. Furthermore, this study highlights those areas of metabolism most likely affected, for future investigations using more targeted metabolomics approaches in relevant biofluids. Besides the small sample size, another limitation of this study is the lack of samples from individuals receiving TB treatment with and without concomitant ART. Examples of possible approaches for future studies include an organic acid extraction and GC–MS, a semi-targeted lipid panel and LC–MS, and positive electrospray ionization mode and derivatization specific for the analyses of fatty acyl derivatives, a targeted acyl-carnitines study using LC–MS, as well as targeted nuclear magnetic resonance spectroscopy for sugars using biofluids from HIV/TB co-infected individuals — with and without treatment (anti-TB drugs and ART). Ideally, from the discussion it is evident that this should be combined with immunological and microbiota studies, as many of these metabolic changes are strongly associated with various immunological responses. Such studies would benefit from concomitant enzymatic assays to obtain more direct mechanistic information (not done here due to limited sample volumes). The host-microbe interactions, including that with communal microbes and the pathogens of interest (i.e., HIV and *Mtb*), at the metabolic level, especially in infectious diseases, is essential to fully understanding the immunometabolic aberrations experienced by the HIV- and *Mtb*-infected host. Such studies may also shed new light on the origins of and mechanisms that sustain the chronic immune activation and inflammation experienced during infection, even when virally suppressed though medication. These studies should ideally be done in a longitudinal study design, in cohorts that are well-characterized clinically and demographically. Furthermore, this study highlighted the need to better characterize the human metabolome, especially with reference to the identification of metabolites that occur in human biofluids but cannot be annotated, as well as those compounds that to date have no known biological function. The ability of metabolomics to distinguish individuals based on their health status was also shown in this investigation, with five supposedly healthy individuals clustering with the samples collected from those with the most advanced disease state. This occurrence would need to be elucidated further and would require metabolomics studies on a population scale to identify if metabolomics can be used to identify a subclinical diseased state that may be present in an individual not yet presenting with symptoms or active disease, for the purpose of early disease detection.

### Supplementary Information

**Additional file 1: Table S1.** Detailed clinical data for HIV-positive cohorts. **Table S2.** Fisher’s exact tests for categorical variables. **Table S3.** Stratification based on CD4 count. **Table S4.** Untreated co-infected group as used for analyses. **Table S5.** t-Test. **Figure S1.** Dendogram of the healthy controls versus untreated HIV/TB co-infected samples, after exclusion of the co-infected samples with high CD4 T-cell counts but before exclusion of any healthy controls. This figure shows that the 5 healthy controls indicated in red cluster with the diseased samples, and were subsequently excluded. **Table S6.** Fold Change. **Table S7.** t-Test. **Table S8.** Fold Change. **Table S9.** t-Test and FC. **Table S10.** PLS-DA. **Figure S2.** The validation results for the PLS-DA analysis of the healthy controls versus all the untreated patient samples. A: Leave-one-out cross-validation results, indicating that PLS-DA with one component is most applicable to these data, B: Permutation testing (frequency = 2000), indicating that the model is not random (significant *p*-value, <0.05). **Table S11.** Results of the ANOVA including all groups (with exclusions of HIV+/TB+/Tn- samples with CD4 T cell count>600 and selected healthy controls). **Table S12.** A count of the compounds significant in the ANOVA post-hoc analysis per compound class. **Table S13.** Results of the ANOVA including all groups (with exclusions of HIV+/TB+/Tn- samples with CD4 T cell count>600) and selected healthy controls, organized by compound class, and then by significance as per ANOVA post-hoc analysis. **Table S14.** The effect of untreated HIV/TB on the metabolome (untreated HIV/TB versus healthy controls). **Table S15.** The effect of HIV on TB (untreated TB vs untreated HIV/TB). **Table S16.** The effect of ART on HIV/TB (untreated HIV/TB versus treated HIV/TB). **Table S17.** Pearson's r correlation results for 3,4-DHBA and all other metabolites (from ANOVA). **Figure S3.** PLS-DA validation results for the comparison of all groups.

## Data Availability

All data that form part of this paper and its supplementary material are free to obtain. Datasets are available on Biostudies, using accession number S-BSST1084.
